# RECIPROCITY AMONG CARE PARTNER DYADS AND ITS INFLUENCE ON OUTCOMES IN DEMENTIA FAMILY CAREGIVERS

**DOI:** 10.1093/geroni/igad104.0957

**Published:** 2023-12-21

**Authors:** Francesca Falzarano, Sara Czaja

**Affiliations:** Weill Cornell Medicine, New York City, New York, United States; Weill Cornell Medicine, New York City, New York, United States

## Abstract

Dementia family caregiving is a heterogenous experience influenced by multidimensional inter- and intra-personal factors. The interdependence between the well-being of caregivers and care-recipients, and that feelings of mutuality and shared involvement in care responsibilities by both the caregiver (CG) and care-recipient (CR) influence family caregiver outcomes. This presentation will present findings from an analysis that examined the influence of caregiver/care-recipient demographic characteristics, care intensity (functional status, medical/nursing tasks), degree of cognitive impairment, and CR depression and quality of life on CG outcomes including burden, self-efficacy, depression, and positive aspects of caregiving. Baseline data were drawn from 99 family caregiver-care-recipient dyads who participated in the Care Partners intervention, a non-pharmacological intervention for individuals with Alzheimer’s disease and their caregivers. Results showed that CG difficulty in performing medical/nursing tasks, greater distress related to the CR’s functional impairment, CR’s degree of cognitive impairment, lower levels of mutuality, and lower CR ratings of quality of life and depression were significantly related to higher CG burden, depression, and lower CG self-efficacy. Alternatively, being a male CG and greater CG distress related to performing medical/nursing tasks and assistance with activities of daily living was associated with lower positive aspects of caregiving. Having a female CR and reporting higher levels of mutuality and preparedness was subsequently associated with more positive appraisals of caregiving. These findings underscore the importance of reciprocal interactions and the need to target both members of the dyad in interventions aiming to improve quality of life and well-being in dementia care partners.

